# Predicting the solubility of CO_2_ and N_2_ in ionic liquids based on COSMO-RS and machine learning

**DOI:** 10.3389/fchem.2024.1480468

**Published:** 2024-10-31

**Authors:** Hongling Qin, Ke Wang, Xifei Ma, Fangfang Li, Yanrong Liu, Xiaoyan Ji

**Affiliations:** ^1^ Energy Engineering, Division of Energy Science, Luleå University of Technology, Luleå, Sweden; ^2^ CAS Key Laboratory of Green Process and Engineering, State Key Laboratory of Mesoscience and Engineering, Beijing Key Laboratory of Ionic Liquids Clean Process, Institute of Process Engineering, Chinese Academy of Sciences, Beijing, China; ^3^ Longzihu New Energy Laboratory, Zhengzhou Institute of Emerging Industrial Technology, Henan University, Zhengzhou, China; ^4^ School of Chemical Engineering, University of Chinese Academy of Sciences, Beijing, China

**Keywords:** ionic liquid, CO_2_ solubility, N_2_ solubility, COSMO-RS, machine learning

## Abstract

As ionic liquids (ILs) continue to be prepared, there is a growing need to develop theoretical methods for predicting the properties of ILs, such as gas solubility. In this work, different strategies were employed to obtain the solubility of CO_2_ and N_2_, where a conductor-like screening model for real solvents (COSMO-RS) was used as the basis. First, experimental data on the solubility of CO_2_ and N_2_ in ILs were collected. Then, the solubility of CO_2_ and N_2_ in ILs was predicted using COSMO-RS based on the structures of cations, anions, and gases. To further improve the performance of COSMO-RS, two options were used, i.e., the polynomial expression to correct the COSMO-RS results and the combination of COSMO-RS and machine learning algorithms (eXtreme Gradient Boosting, XGBoost) to develop a hybrid model. The results show that the COSMO-RS with correction can significantly improve the prediction of CO_2_ solubility, and the corresponding average absolute relative deviation (AARD) is decreased from 43.4% to 11.9%. In contrast, such an option cannot improve that of the N_2_ dataset. Instead, the results obtained from coupling machine learning algorithms with the COSMO-RS model agree well with the experimental results, with an AARD of 0.94% for the solubility of CO_2_ and an average absolute deviation (AAD) of 0.15% for the solubility of N_2_.

## 1 Introduction

Since the dawn of the industrial revolution, the rising consumption of fossil fuels has caused a significant increase in atmospheric carbon dioxide (CO_2_) levels. The worldwide atmospheric CO_2_ levels have increased from an average of 280 parts per million (ppm) in the late 18th century to 414 ppm by the year 2021 (Cheng et al., 2022). As a consequence, this rise has triggered numerous environmental challenges, such as global warming and the acidification of oceans. Mitigating CO_2_ emissions is thus crucial. Meanwhile, CO_2_ serves as an inexpensive, non-toxic, and abundant C1-feedstock, and it can be converted into alcohols, ethers, acids, and various other value-added chemicals. Therefore, CO_2_ capture and utilization via conversion is one of the effective strategies to mitigate CO_2_ emission and produce carbon-based chemicals.

Among different CO_2_ conversion methods, the electrochemical CO_2_ reduction reaction (eCO_2_RR) stands out as an appealing strategy to convert renewable electricity, together with CO_2_, into fuels and feedstocks in the form of chemical bonds (Vasileff et al., 2018). Notably, the electrochemical synthesis of compounds with C-N bonds, such as urea, amide, and amino acids, from CO_2_ and N_2_ as well as their derivatives is gaining recognition as a viable and sustainable approach (Chen et al., 2020a; Jouny et al., 2019). Additionally, nitrogen (N_2_), comprising 78% of the atmospheric air, is a highly appealing source of nitrogen. Consequently, the electrocatalytic N_2_ reduction reaction (NRR) for ammonia production has attracted substantial attention for its advantages in energy conservation and environmental sustainability. Despite considerable progress, the low solubility of CO_2_ and N_2_ in water and conventional electrolyte solutions leads to low efficiency of the aforementioned reactions, thus hindering its development and application (Chen et al., 2021; Ren et al., 2021). Hence, for both eCO_2_RR with C-N coupling and NRR mentioned above, enhancing the solubility of CO_2_ and N_2_ is a vital prerequisite for the subsequent conversion reaction.

For eCO_2_RR and NRR, the gas solubility can be adjusted by developing novel electrolytes. Ionic liquids (ILs) are a type of organic salt that remains liquid at or near room temperature and consists of cations and anions. As a kind of green medium, they possess many outstanding characteristics, such as flexible tunability, high ionic conductivity, and wide electrochemical window. ILs have been extensively studied and shown great potential in many fields, such as electrocatalytic conversion, over the past decade. For example, the work by Chen et al. demonstrated that the faradaic efficiency (FE) and current density in 0.5 M [Bmim] PF_6_/MeCN for CO_2_ electrochemical reduction to CO are much higher than those in 0.5 M KHCO_3_, and the high CO_2_ solubility of [Bmim][PF_6_]/MeCN is one of the reasons (Chen et al., 2020b). Zhou et al. studied the electrochemical ammonia synthesis at ambient conditions and achieved a FE of NRR higher than 60%. A key factor in this high efficiency was the relatively elevated N_2_ solubility in the IL electrolyte (Zhou et al., 2017). Therefore, using IL as electrolytes can be an effective strategy to enhance the gas solubility and thus improve the performance of eCO_2_RR and NRR.

ILs can be theoretically composed of any combination of cations and anions, making ILs highly desirable but also time-consuming and expensive to measure their properties experimentally. Therefore, a fast and reliable predictive method is needed to screen out the suitable ILs for specific tasks, such as finding ILs with high gas solubility for electrocatalytic conversion of CO_2_ and N_2_. Several models have been developed and applied to predict the solubility of gases in the systems containing ILs. Molecular dynamics (MD) simulations, frequently combined with density functional theory (DFT), provide valuable microscopic insights and serve as a robust complement to experimental results (Zhao et al., 2024). While these computational techniques have significantly enhanced our understanding of ILs properties, their limitations such as complex model architectures and extended computational times have constrained the efficiency and broader application of these methods in IL research. In addition, the activity coefficient models, such as UNIFAC (Chen et al., 2020c), UNIQUAC, (Kamgar and Rahimpour, 2016) etc., usually show good capabilities in predicting the solubility of gases in ILs. However, these models require parameters of each functional group and the binary interaction coefficients among them, and their application is limited to a certain extent. The methods based on quantum chemistry (QM) overcome the limitations of the aforementioned techniques by obtaining missing molecular properties through *ab initio* calculations, being independent of experimental data. Furthermore, some QM-based methods have already been applied to Computer-Aided Molecular Design (CAMD) methods, such as those based on the Conductor-like Screening Model (COSMO), including the Conductor-like Screening Model for Realistic Solvents (COMSO-RS) proposed by Klamt (1995) and the COSMO segment activity coefficient (COMSO-SAC). Ali et al. employed COSMO-RS to predict the solubility of CO_2_ in eight different ILs. The predictions were then compared to experimental data, showing similar trends and a moderate level of agreement, with deviations ranging from 8% to 62% (Hadj-Kali et al., 2020). The CO_2_ absorption capacity of 1,2,4-triazolium-based ILs and the imidazolium-based ILs with different anions was predicted with COSMO-RS, and the triazolium-based ILs exhibited higher values (Mohammed et al., 2023). It was also found that the HOMO energy level of the anion plays a more prominent role in solubility compared to the LUMO energy level of the cation, which can be explained by the greater tendency of CO_2_ to accept electrons more rather than donate them. Manan et al. verified the predictive accuracy of COSMO-RS by investigating the solubility of 15 gases, including CO_2_ and N_2_, in 27 different ILs. The study demonstrated that, while COSMO-RS can qualitatively predict solubility, its accuracy needs to be further improved for reliable quantitative predictions. For example, the absolute relative deviations (ARD) of the CO_2_ solubility in [Bmim][BF_4_] is as high as 32.4% and that for N_2_ is 57.8% (Manan et al., 2009). A common method to improve the prediction performance of COSMO-RS is to employ experimental data to correct the model predictions. For instance, Zhao et al. (2017), Liu et al. (2021), Wang et al. (2021), and Farahipour et al. (2016) used a linear expression to correct the Henry’s law constants obtained from COSMO-RS. However, no work has been done so far to study a wide range of ILs, and the work is on the CO_2_ solubility but not on the N_2_ solubility.

In recent years, benefiting from the rapid development of machine learning algorithms, quantitative structure-property relationship (QSPR) models have been extensively applied to predict the properties of ILs, such as density, viscosity, activity coefficient, gas solubility, and so on. Song et al. (2020) employed the artificial neural network (ANN) and support vector machine (SVM) algorithms to construct predictive models based on group contribution (GC) methods, effectively predicting the solubility of CO_2_ in various ILs using 10,116 datasets across different temperatures and pressures. The ANN-GC model has an estimated mean absolute error (MAE) of 0.0202 and a coefficient of determination (R^2^) of 0.9836, while the SVM-GC model shows a MAE of 0.0240 and a R^2^ of 0.9783. Tian et al. integrated ANN and SVM with the ionic fragments contribution (IFC) to predict the solubility of CO_2_ and N_2_ in ILs. In their work, 13,055 datasets of CO_2_ solubility and 415 datasets of N_2_ solubility were collected for model training and validation. As a result, the R^2^ values obtained for the CO_2_ solubility predictions are 0.9855 for IFC-SVM and 0.9732 for IFC-ANN in the training sets. Similarly, the R^2^ values for the N_2_ solubility predictions are 0.9966 and 0.9909 for IFC-SVM and IFC-ANN, respectively (Tian et al., 2023). Recently, Tian et al. established two models based on both the random forest (RF) and gradient boosting regressor (GBR) to predict the N_2_ solubility in ILs. The input features of the model include temperature, pressure, and COSMO-derived descriptors. After training the model with four of five folders, R^2^ and AARD were obtained with values of 0.9986% and 14.24% for RF-IFC and 0.9999% and 5.28% for GBR-IFC, respectively (Tian et al., 2024). Ali and co-workers developed two deep learning models, namely, ANN and long short-term memory (LSTM), to predict CO_2_ solubility in ILs using a dataset of 10,116 data points across 164 kinds of ILs under various temperature and pressure conditions. Both models demonstrated strong predictive performance, with R^2^ values of 0.986 and 0.985 for ANN and LSTM, respectively. Moreover, the results showed that while both models provided excellent accuracy in predicting CO_2_ solubility, the ANN model achieved reliable accuracy with significantly lower computational time compared to the LSTM model (Ali et al., 2024). The above results confirm that the prediction models originated from the GC methods combined with the ML algorithms can be used to predict the solubility of CO_2_ and N_2_ effectively.

However, to the best of our knowledge, it was found that there are only a few research using COSMO-RS to predict the solubility of N_2_ in ILs, and its prediction capacity of COSMO-RS is uncertain. In addition, there is a lack of robust models to predict the gas solubilities based on COSMO-RS that already qualitatively represent the gas solubility. Hence, in this work, the solubility of CO_2_ and N_2_ in various ILs over wide ranges of temperature and pressure was extensively studied based on COSMO-RS. Firstly, a comprehensive collection of the literature data on the solubility of CO_2_ and N_2_ in ILs was conducted. Subsequently, COSMO-RS was utilized to predict the solubility of CO_2_ and N_2_ in ILs, accompanied by discussion and analysis. To further improve the performance of COSMO-RS, the modification was carried out, including two options: a correction method and a hybrid model based on the ML algorithm and GC method.

## 2 Modelling

### 2.1 COSMO-RS

All COSMO-RS calculations were performed using the COSMOtherm software (version 19.0.4, with the BP_TZVP_19.ctd parameterization, COSMOlogic, Leverkusen, Germany). To begin with, the quantum chemical Gaussian09 package was employed to optimize the structures of the studied compounds, which include CO_2_, N_2_, and components of IL, at the B3LYP/6-31++G (d, p) level. Frequency calculations were conducted to confirm that the optimized structures correspond to true minima. Second, the resulting COSMO files of the optimized structures were subsequently imported into the COSMOtherm program to compute the solubility of CO_2_ and N_2_ in the studied ILs. For the solubility calculations of gases in ILs, the cation and anion components are treated as separate molecules with equal molar fractions (n_cation_ = n_anion_ = n_IL_), furthermore, the input variables (*T*, *P*) were set to be consistent with the experimental conditions reported in the literature.

### 2.2 Machine learning

At present, multiple ML algorithms have been used to estimate the physical and thermodynamic properties of ILs and IL-involved systems. Among them, the XGBoost algorithm proposed by Chen and Guestrin (2016) is a powerful and efficient algorithm owing to its high training efficiency, good prediction effect, multi-controllable parameters, and user-friendly features. XGBoost can be regarded as a variant of Gradient Boosting Decision Tree (GBDT). Unlike GBDT, XGBoost introduces regular terms to limit the model complexity to reduce the probability of over-fitting, and the second-order derivative information is used for optimization, which accelerates the convergence process of the model and improves the training efficiency. By assuming a dataset contains *n* examples and *m* features, the mathematic expressions (Equation 1) and objective function (Equation 2) of the XGBoost algorithm are outlined as follows:

$${\hat{y}}_{i}=\sum_{k=1}^{K}f_{k}\left(x_{i}\right),f_{k}\in F$$ (1)

Here, *f*_*k*_ is the *k*th independent tree, and *F* represents the space of regression trees.

$$obj=\sum_{i=1}^{n}l\left({\hat{y}}_{i}{,y}_{i}\right)+\sum_{k=1}^{N}\Omega \left(f_{k}\right)$$ (2)

where *l* is a differentiable convex loss function that measures the difference between the prediction ${\hat{y}}_{i}$ and the target $y_{i}$ , and *Ω* is the regularization term.

### 2.3 Hybrid model

Since the selection and number of features (i.e., the functional groups) significantly affect the accuracy and generalization ability of the ML model, the division of the functional groups followed the JR method in this work (Nannoolal et al., 2007), with the detailed information provided in Supplementary Table S1. Also, for the studied ILs, the same functional group may be contained in both cations and anions, and to better describe the impact of functional groups in anions and cations on solubility, a “-” sign was added after the functional groups from anions. Consequently, the studied ILs were divided into 41 groups for CO_2_ solubility modeling and 38 groups for N_2_ solubility modeling.

Before model development, the data used were normalized and standardized to eliminate the effects of data magnitude. First, the CO_2_ and N_2_ solubility datasets were divided into the training set and the test set, with a division ratio of 8:2. The input features for the XGBoost-GC model include temperature (*T*), pressure (*P*), and groups on cations and anions (41 for CO_2_ dataset, and 38 for N_2_ dataset). The target variable for the CO_2_ dataset was set to be the relative deviation ( $\frac{x_{\mathrm{Exp}}-x_{COSMO-RS}}{x_{\mathrm{Exp}}}$ ) between the experimental results and the predictions generated by the original COSMO-RS model. For N_2_, the target variable is the absolute deviation ( $x_{\mathrm{Exp}}-x_{COSMO-RS}$ ) between the experimental values and the COSMO-RS model predictions for each sample. For comparison, a model with the same input features but using experimental values as the target variables was also studied, which is named XGBoost-GC-D.

The optimal parameters were obtained through the Bayesian optimization algorithm. Since the XGBoost algorithm is a decision tree-based model, the number of trees should be proper, and too few trees will result in poor prediction, while too many trees may lead to over-learning and over-fitting. The same goes for the maximum depth of the tree. Therefore, simultaneous optimization was performed on these parameters, where the number of trees ranged from 1 to 100 with corresponding maximum depth from 1 to 10. The ranges for the learning rate and subsample ratio were set to 0.01–0.3 and 0–1, respectively. The number of iterations was 200, and the specific parameters are listed in Supplementary Table S2.

## 3 Results and discussion

### 3.1 Data collection

Given that the work of Lei et al. (2014) systematically collected CO_2_ solubility data in ILs published before 2013 and used it as a database, the CO_2_ solubility data used in this work mainly come from literature reported in the past decade (Nonthanasin et al., 2014; Tagiuri et al., 2014; Gonzalez-Miquel et al., 2014; Makino et al., 2014a; Bahadur et al., 2015; Carvalho et al., 2014; Makino et al., 2014b; Zhou et al., 2014; Zeng et al., 2015; Almantariotis et al., 2017; Liu et al., 2016; Zhou et al., 2016; Zoubeik et al., 2016; Nematpour et al., 2016; Watanabe et al., 2016; Zubeir et al., 2016a; Zubeir et al., 2016b; Dai et al., 2017; Jalili et al., 2017; Bai et al., 2017; Mirzaei et al., 2018; Zhao et al., 2018; Jalili et al., 2019; Nath and Henni, 2020; Safarov et al., 2019; Wang et al., 2022; Henni et al., 2023; Kodama et al., 2023; Mirzaei et al., 2023; Suzuki et al., 2024a; Suzuki et al., 2024b), and the experimental data with zero or negative solubility are not considered. Finally, 3,036 sets of CO_2_ solubility (mole fraction: 0.00116–0.713) in 72 different ILs were selected at temperatures of 273.15–413.15 K and pressures of 9.7–6,532.8 kPa.

However, for N_2_, the relevant experimental data are much less abundant than for CO_2_. Here, we collected and screened N_2_ solubility data in the previous literature (Zhou et al., 2014; Almantariotis et al., 2017; Liu et al., 2016; Zhou et al., 2016; Jacquemin et al., 2006a; Jacquemin et al., 2006b; Zhou et al., 2013; Almantariotis et al., 2012; Stevanovic and Gomes, 2013; Zhao et al., 2011; Anderson et al., 2007; Yuan et al., 2006; Afzal et al., 2015; Zhang et al., 2017; Bentley et al., 2023). Similarly, the datasets with zero or negative solubility were discarded. A total of 457 N_2_ solubility data points in 31 types of ILs were collected, with values ranging from 0.000171 to 0.6187 mol fraction at 283.20–353.20 K and 4.69–14982 kPa. Supplementary Tables S3, S4 provided the detailed experimental ranges of temperature, pressure, and solubility for various CO_2_-IL and N_2_-IL systems.

Supplementary Figures S1, S2 show the temperature, pressure, and solubility distributions. It could be seen that the temperature data distribution of the two datasets is relatively uniform, while the pressure data were mainly concentrated in 0–1,000 kPa. The CO_2_ solubility data is relatively evenly distributed, while the N_2_ solubility data is mainly concentrated below 0.05.

The chemical structures of the cations and anions investigated in this work are illustrated in Supplementary Table S5. The cations include imidazolium, pyridinium, pyrrolidinium, ammonium, and phosphonium, and the anions contain acetate, sulfate, sulfonate, tetrafluoroborate [BF_4_], hexafluorophosphate [PF_6_], Bis [(trifluoromethyl)sulfonyl]azanide [NTf_2_], etc.

### 3.2 Model performance

Appropriate model evaluation metrics are crucial for evaluating the accuracy of the model. To provide a reasonable evaluation, the average absolute relative deviation (AARD, Equation 3) and coefficient of determination (R2, Equation 4) were used to quantify the discrepancies between the experimental and predicted CO_2_ solubilities, where the former is a bias-centric metric while the latter is a variance-oriented one. However, for the N_2_ dataset, considering the low accuracy of experimental measurements linked to the low solubility of N_2_ in the solvents, the verage absolute deviation (AAD, Equation 5) and R^2^ were used.

$$AARD\%=\frac{1}{N}\sum_{i=1}^{N}\left|\frac{\left(x_{i}-x_{i}^{\prime }\right)}{x_{i}}\right|\times 100$$ (3)

$$R^{2}=1-\frac{\sum_{i=1}^{N}{\left(x_{i}^{\prime }-x_{i}\right)}^{2}}{\sum_{i=1}^{N}{\left({\bar{x}}_{i}-x_{i}\right)}^{2}}$$ (4)

$$AAD\%=\frac{1}{N}\sum_{i=1}^{N}\left|x_{i}-x_{i}^{\prime }\right|\times 100$$ (5)

where *N* is the total number of samples, the experimental and predicted values of gas solubility in ILs are denoted as $x_{i}$ and $x_{i}^{\prime }$ , respectively, and ${\bar{x}}_{i}$ represents the mean value of the gas solubility in ILs.

### 3.3 COSMO-RS predictions

**FIGURE 1:**
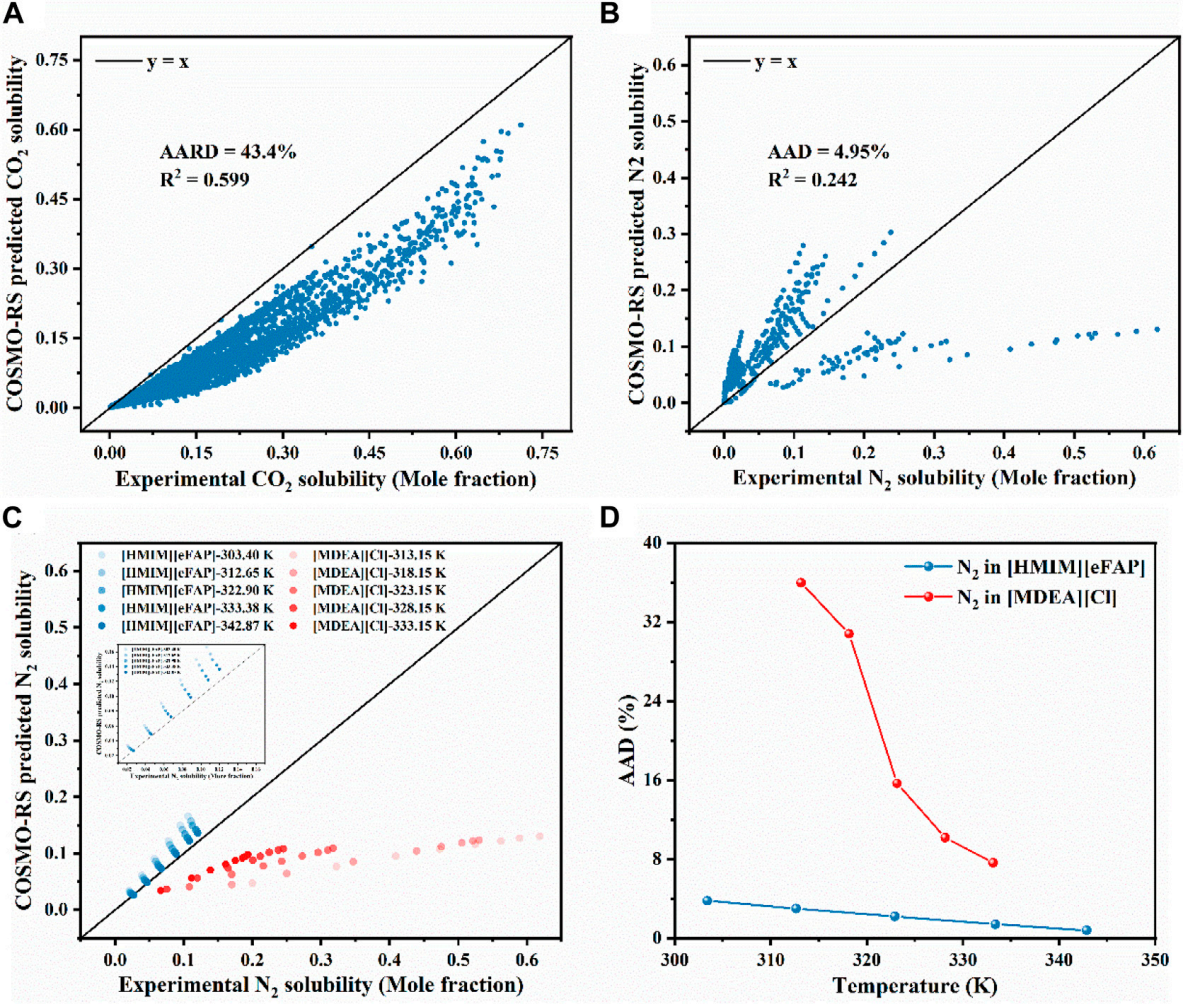
Comparison of experimental and COSMO-RS predicted solubility for **(A)** CO_2_ and **(B)** N_2_ in various ILs, **(C)** Comparison of experimentally determined and COSMO-RS predicted N_2_ solubility in [HMIM][eFAP] and [MDEA][Cl], **(D)** AAD of COSMO-RS model predictions at different temperatures.

As described in Section 2.1, the solubility of CO_2_ and N_2_ in the identified ILs under the same conditions (*T*, *P*, ILs) as reported in the literature was predicted using COSMOthermX (version 19.0.4) and compared with the experimental values (see Supplementary Tables S6, S7). Figures 1A, B present the comparison of the experimentally determined and COSMO-RS predicted gas solubility of CO_2_ and N_2_, respectively. In Figure 1A, it is evident that the COSMO-RS model tends to underpredict the solubility of CO_2_ in ILs, with an AARD of 43.4% and a R^2^ of 0.599. For N_2_, as depicted in Figure 1B, the solubility data are spread on either side of the diagonal, with an AAD of 4.95% and a R^2^ of 0.242.

It should be emphasized that the overall trend of the solubilities predicted by COSMO-RS is consistent with the experimental data at various temperatures and pressures (Supplementary Figures S3, S4), confirming that the qualitative prediction of COSMO-RS can be used to reliably screen ILs based on their gas solubilities. To further analyze the model predictions, the N_2_ solubility in two ILs was taken as an example to discuss the effects of temperature and pressure. As depicted in Figure 1C, the model prediction performance for [HMIM][eFAP] depends on the studied temperature. As the temperature increase, the points on the consistency diagram get closer to the diagonal line, i.e., the model prediction gets close to the experimental results, and thus the corresponding AAD gradually decreases. This indicates that the prediction of COSMO-RS is more accurate at relatively high temperatures. The same trend was observed for [MDEA][Cl] (Figures 1C, D). Additionally, when the temperature remains constant (e.g., *T* = 303.4 K), as the pressure increases, both the experimentally measured and theoretically predicted solubilities of N_2_ in [HMIM][eFAP] show the same increasing trend and the accuracy of COSMO-RS is gradually decreasing (Supplementary Figure S5). The results of this study demonstrate that, within a certain temperature and pressure range, COSMO-RS can accurately capture the effects of input variables (*T, P*) on the solubility of N_2_ in different cation and anion combinations.

Furthermore, the results of the COSMO-RS model developed in this study were compared with other predictive models reported in the literature. For example, Kamgar and Rahimpour (2016) used UNIQUAC and quantum models to predict the solubility of CO_2_ in seven ILs. The study found that UNIQUAC showed good prediction ability for the ILs studied, the ARD in most cases lower than 5%, and the maximum ARD is 9.17%. The predictions of the UNIQUAC model in the literature perform better. Additionally, the COSMO-RS model was also used to predict the CO_2_ solubility for the same system, showing an ARD ranging from 6.1% to 62.4%, especially, when the pressure increases, the error becomes larger. Recently, Chen and co-workers used a hierarchical extension strategy to develop a UNIFAC-IL-Gas model for gas solubility prediction. The results showed that for 13 types of gases, including CO_2_ and N_2_, its prediction performance exceeded the COSMO-RS model (Chen et al., 2020c). The above results further confirm that compared to models that require parameters obtained from the fitting of experimental data, the COSMO-RS model without requirements of any experimental information predicts results qualitatively.

### 3.4 COSMO-RS correction

As mentioned before, many studies have demonstrated that higher accuracy can be achieved by performing linear regression on the predicted values obtained by COSMO-RS. These corrected models typically use the experimental values as the target variables. However, in this work, it is evidenced that there is no simple linear relationship between *T*, *P*, $x_{COSMO-RS}$ and $x_{\mathrm{Exp}}$ (Supplementary Figures S6, S7), a polynomial expression (Equation 6) combined with different regression strategies were used to further improve the prediction of COSMO-RS:

$$∆x=f\left(T,P\right)$$ (6)

For CO_2_, the relative deviation ( $∆x_{1}=\frac{x_{\mathrm{Exp}}-x_{COSMO-RS}}{x_{\mathrm{Exp}}}$ ) was used (Equation 7):

$$∆x_{1}=k_{1}T+k_{2}P+k_{3}T^{2}+k_{4}TP+k_{5}P^{2}$$ (7)

For N_2_, the absolute deviation ( $∆x_{2}=x_{\mathrm{Exp}}-x_{COSMO-RS}$ ) was used (Equation 8):

$$∆x_{2}=k_{1}T+k_{2}P+k_{3}T^{2}+k_{4}TP+k_{5}P^{2}$$ (8)

Here, k_1_-k_5_ are the adjustable parameters.

**FIGURE 2:**
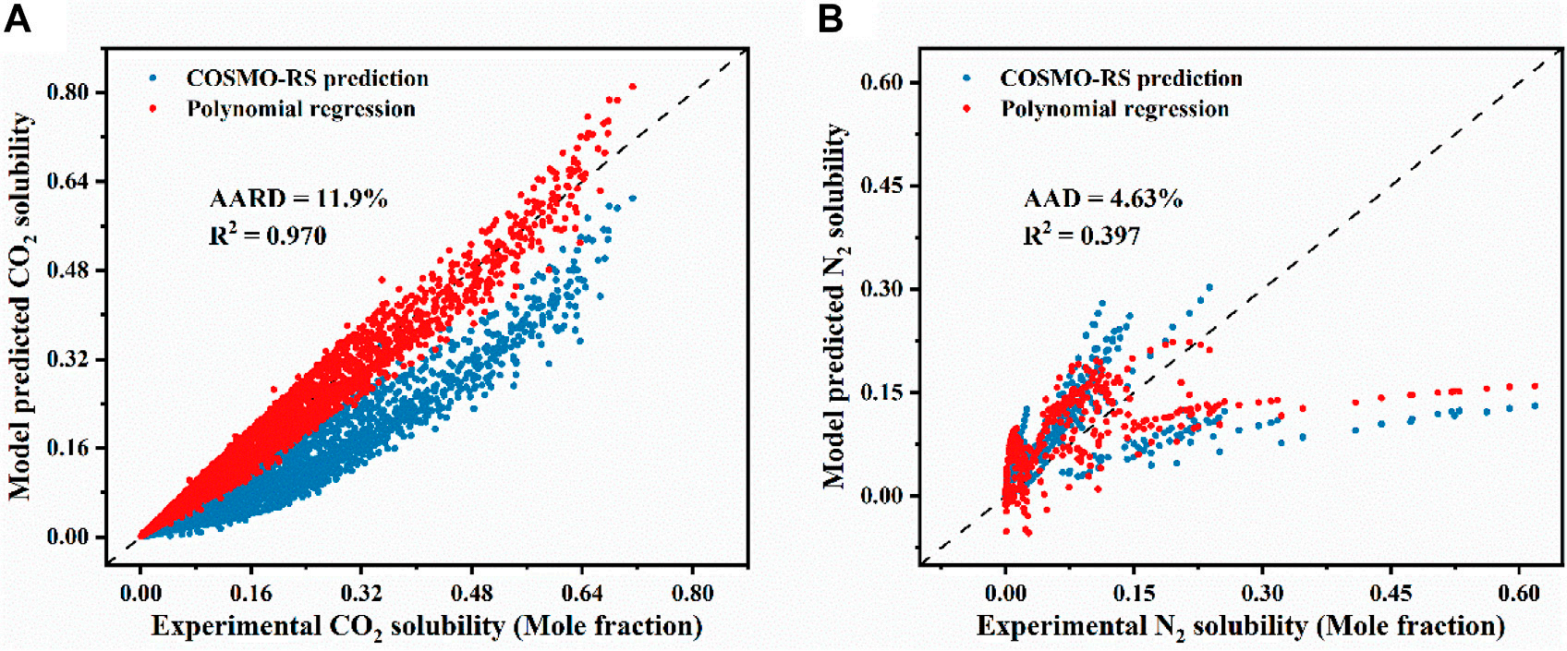
Comparison of experimental and model-predicted solubility of **(A)** CO_2_ and **(B)** N_2_ in various ILs.

Based on the collected data point, the adjustable parameters were obtained, as listed in Supplementary Table S8. Figure 2 shows the comparison between the experimental gas solubilities and those predicted by the two models. It can be evident from Figure 2A that the CO_2_ solubility predictions from the modified model align more closely with the experimental values than those from the original COSMO-RS model. After the model modification, its AARD was decreased to 11.9% with a R^2^ of 0.970. For comparison, the AARD for the original COSMO-RS is 43.4%. For N_2_, the modified model shows only a very slight decrease in AAD, and there is no noticeable improvement in R^2^ compared with that before the modification. These results demonstrate that the corrected model improves the accuracy of the COSMO-RS model for predicting CO_2_ solubility. However, such a correction does not work for the solubility of N_2_ in ILs. The reasons for the above phenomenon are summarized as follows: 1) The CO_2_ dataset and the N_2_ dataset may have different quality levels. The data in the CO_2_ dataset is more accurate and complete and thus can be corrected for better accuracy. 2) The model assumptions themselves and the selection of features are less applicable to the N_2_ dataset than to the CO_2_ dataset. 3) The insufficient number of samples in the N_2_ dataset prevents the model from effectively learning the relationship between the initial predicted value and the experimental value.

### 3.5 Hybrid models

The COSMO-RS model can be used for qualitative prediction, which is sufficient for IL screening. The correction with a polynomial expression on COSMO-RS can improve the prediction capability in the solubility for certain gases (CO_2_, etc.) but not for all (e.g., N_2_). In this section, an alternative option was used to develop a hybrid model, where XGBoost-GC was coupled with COSMO-RS to achieve reliable predictions of CO_2_ and N_2_ solubility in ILs.

#### 3.5.1 CO_2_ solubility

**FIGURE 3:**
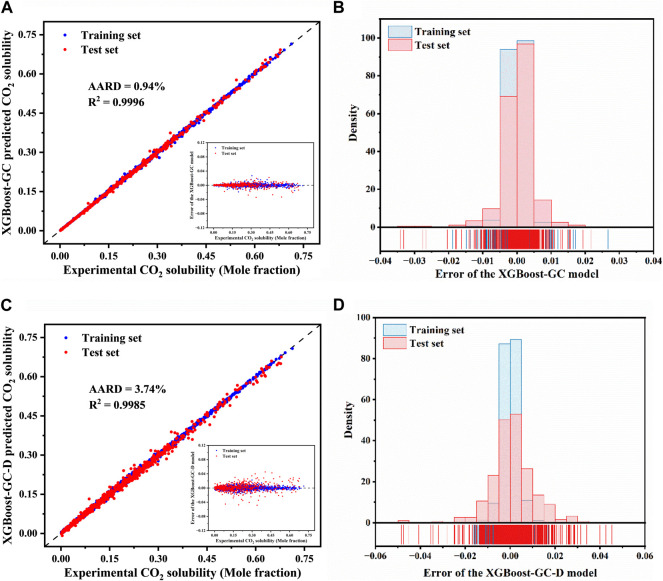
Comparison of experimental CO_2_ solubility in ILs with predictions from **(A)** the XGBoost-GC and **(C)** the XGBoost-GC-D models, (The inset shows the prediction errors for CO_2_ solubility by the XGBoost-GC model and XGBoost-GC-D model). Distribution of prediction errors for CO_2_ solubility as predicted by **(B)** the XGBoost-GC model and **(D)** the XGBoost-GC-D model.

The comparison between experimentally determined and XGBoost-GC model-predicted CO_2_ solubility for both the training and test sets is depicted in Figure 3A, with the detailed data listed in Supplementary Table S9. Unlike the corrected COSMO-RS model (as seen in Figure 2A), the XGBoost-GC model demonstrates a significantly better alignment with the diagonal, indicating an improved prediction accuracy. The AARD for the entire dataset is as low as 0.94%, with a R^2^ of 0.9996. In comparison, the XGBoost-GC-D model, which directly uses experimental values as target variables, also shows good prediction capabilities, achieving an AARD of 3.74% and an R^2^ of 0.9985. This performance may be due to the meticulous division of IL groups and the optimization of the model Hyperparameter.

For a thorough evaluation of the model predictions, the discrepancies between experimental and model-predicted CO_2_ solubilities are plotted against the experimental values (refer to the inset in Figure 3A). The error distribution is also displayed in Figure 3B. It is clear that the majority of the errors are closely clustered around zero, signifying a high degree of accuracy for the XGBoost-GC model, with only a small fraction of errors exceeding ± 0.03. These larger errors tend to occur when the solubility of CO_2_ exceeds 0.3, with the maximum absolute error being approximately −0.034. On the other hand, the error distribution for the XGBoost-GC-D model (Figure 3D) exhibits a more disordered pattern, with errors distributed across a wider range, and the maximum error is −0.049. This suggests that the XGBoost-GC-D model is less accurate compared to XGBoost-GC. Therefore, it can be concluded that the XGBoost-GC model provides more accurate predictions, making it the more reliable hybrid model for predicting CO_2_ solubility.

We further compared the performance of the established model with those reported in the literature. The detailed statistical results are shown in Table 1. To predict the CO_2_ solubility, regardless of whether the input features are group information or other descriptors, the hybrid model XGBoost-GC achieved higher prediction accuracy with less data, reflecting the superior performance of the XGBoost-GC model.

**TABLE 1:** Comparison of the models established in this work and reported in the literature for CO_2_ solubility prediction.

Model	Total data points	R^2^	AARD	AAD (MAE)	References
XGBoost-GC	3,036	0.9996	0.94%	0.00146	This work
XGBoost-GC-D	3,036	0.9985	3.74%	0.00333	This work
ANN-GC^a^	10,116	0.9836	-	0.0202	Song et al. (2020)
SVM-GC^a^	10,116	0.9783	-	0.0240	Song et al. (2020)
IFC-SVM^a^	13,055	0.9763	-	0.0192	Tian et al. (2023)
IFC-ANN^a^	13,055	0.9711	-	0.0261	Tian et al. (2023)
SE-MLP	9,224	0.9873	-	0.0169	Liu et al. (2023)
ANN	2,930	0.9947	3.58%	-	Sedghamiz et al. (2015)

^a^ represents the data from the test set.

#### 3.5.2 N_2_ solubility

**FIGURE 4:**
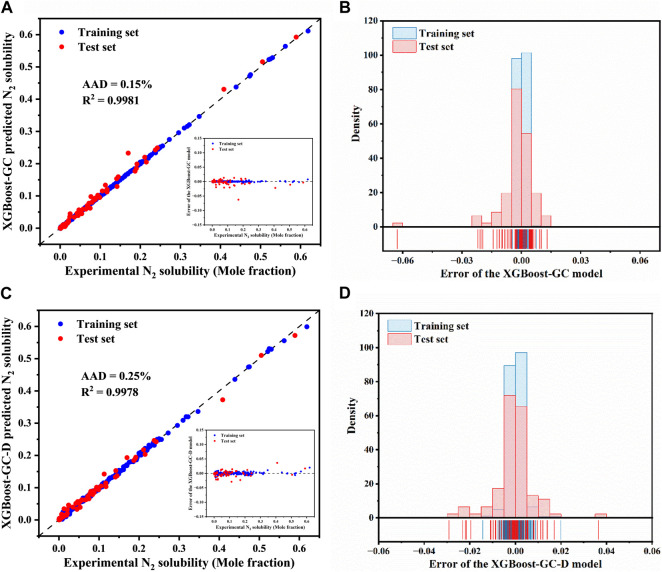
Comparison of experimental N_2_ solubility in ILs with predictions from **(A)** the XGBoost-GC and **(C)** the XGBoost-GC-D models, (The inset shows the prediction errors for N_2_ solubility by the XGBoost-GC model and XGBoost-GC-D model). Distribution of prediction errors for N_2_ solubility as predicted by **(B)** the XGBoost-GC model and **(D)** the XGBoost-GC-D model.

The experimentally determined and ML model-predicted N_2_ solubility for the both training and test sets are illustrated in Figures 4A, C, detailed data are provided in Supplementary Table S10. It can be clearly observed from Figure 4A that the majority of data points, for both the training and test sets, are closely aligned along the *y* = *x* line, indicting high accuracy in the predictions of the XGBoost-GC model. The model achieved an R^2^ of 0.9981 and an AAD of 0.15% across the entire dataset, demonstrating significant improvement in the predictions of the hybrid model over the original COSMO-RS model. Similarly, the XGBoost-GC-D model also exhibits good predictive performance, though slightly less accurate than the XGBoost-GC model. As shown in Figures 4A, B, the majority of the errors for the XGBoost-GC model fall within the range of ± 0.02, with the maximum absolute error being around −0.062. In contrast, Figure 4D illustrates that most of the errors for the XGBoost-GC-D model are close to zero, although a few errors exceed ± 0.03, with the maximum reaching approximately 0.036. This discrepancy could potentially be due to the limited amount of available data, highlighting the importance of conducting more experimental measurements to improve the robustness of the model.

Table 2 summarizes a comparison of different models, mainly including IFC-SVM, IFC-ANN, RF-IFC and GBR-IFC. The table shows that when the amount of data and the number of ILs are both similar, the R^2^ and AAD of the XGBoost-GC model are better than those of the SVM-IFC, ANN-GC, and RF-IFC models proposed by Tian et al., but not as good as the GBR-IFC model. This may be attributed to the fact that they introduced COSMO-derived descriptors as input variables, which contain more molecular information such as electronic distribution, molecular size, etc., making the input parameter information more comprehensive and thus achieving higher prediction accuracy.

**TABLE 2:** Comparison of the models established in this work and reported in the literature for N_2_ solubility prediction.

Model	Total data points	Total ILs	R^2^	AAD (MAE)	References
XGBoost-GC	457	31	0.9981	0.0015	This work
XGBoost-GC-D	457	31	0.9978	0.0025	This work
IFC-SVM^a^	415	38	0.9829	0.00620	Tian et al. (2023)
IFC-ANN^a^	415	38	0.9886	0.00560	Tian et al. (2023)
RF-IFC	385	38	0.9986	0.00188	Tian et al. (2024)
GBR-IFC	385	38	0.9999	0.000123	Tian et al. (2024)

^a^ represents the data from the test set.

### 3.6 Challenges and prospects

Machine learning has demonstrated significant potential in predicting various properties of ILs, particularly in fields such as green chemistry and electrochemical processes. ILs possess a variety of tunable properties, which are often time-consuming and costly to determine experimentally. ML models, trained on experimental data or theoretical predictions, offer a rapid and efficient means of predicting key properties such as viscosity, density, conductivity, and solubility. However, the performance of ML models is highly dependent on the quality and comprehensiveness of the datasets used for training, and thus the availability of high-quality data remains a critical challenge.

In addition, various thermodynamic models have shown high prediction accuracy for IL-containing systems due to their solid thermodynamic foundations. Effectively combining ML algorithms with these models to improve prediction accuracy without relying on large amounts of experimental data is crucial yet highly challenging.

The accuracy of ML models is highly depended on the selection of meaningful features, such as temperature, pressure, and structural information. The selection of features that better represent the geometric and electronic structures of ILs, along with the application of data-cleaning techniques, can further improve prediction accuracy. Additionally, future advancements may involve the implementation of more sophisticated algorithms, such as deep neural networks, which have the potential to capture complex, non-linear relationships between the structures of ILs and their corresponding properties.

## 4 Conclusion

Ionic liquids (ILs) are an emerging category of chemicals that have shown promise as electrolytes or co-catalysts for CO_2_ and N_2_ electrocatalytic conversion. The combination of cations and anions makes it highly designable but also presents a significant challenge in screening out suitable ILs for specific tasks. In this work, we developed different strategies based on the COSMO-RS model to accurately predict the CO_2_ and N_2_ solubility, thus aiding in the screening of the optimal ILs for the electrocatalytic conversion of CO_2_ and N_2_. We first established a database containing 3,036 solubility data for CO_2_ and 457 solubility data for N_2_ in ILs at various temperatures and pressures. The COSMO-RS model was employed to predict the solubility of CO_2_ and N_2_. The AARD between the experimental and COSMO-RS predicted solubilities of the CO_2_ was relatively high, i.e., 43.4% and the R^2^ for the CO_2_ and N_2_ datasets are 0.599 and 0.242, respectively. Polynomial regression was employed to correct the COSMO-RS predicted solubilities, resulting in a significant decrease in AARD for CO_2_ and a slight decrease in AAD for N_2_. Further performance improvements were achieved through a hybrid model that combined COSMO-RS with machine learning and group information methods. The developed hybrid model demonstrated better prediction performance, with high R^2^ and low AARD for the CO_2_ dataset and low AAD for the N_2_ dataset.

## Data Availability

The datasets presented in this study can be found in online repositories. The names of the repository/repositories and accession number(s) can be found in the article/Supplementary Material.
