# Supplementary material for: Predicting the solubility of CO2 and N2 in ionic liquids based on COSMO-RS and machine learning
Source: Front Chem. 2024 Oct 31;12:1480468. doi: 10.3389/fchem.2024.1480468 (PMC11560425; doi:10.3389/fchem.2024.1480468)
Supplement: Supplementary file 2 [file DataSheet1.docx]

Predicting the solubility of CO_2_ and N_2_ in ionic liquids based on COMSO-RS and machine learning

Hongling Qin^1,2^, Ke Wang^2,3^, Xifei Ma^2,4^, Fangfang Li^1^, Yanrong Liu^2,3,4 *^, Xiaoyan Ji^1, *^

^1^Energy Engineering, Division of Energy Science, Luleå University of Technology, 97187 Luleå, Sweden

^2^CAS Key Laboratory of Green Process and Engineering, State Key Laboratory of Mesoscience and Engineering, Beijing Key Laboratory of Ionic Liquids Clean Process, Institute of Process Engineering, Chinese Academy of Sciences, Beijing 100190, China

^3^Longzihu New Energy Laboratory, Zhengzhou Institute of Emerging Industrial Technology, Henan University, Zhengzhou 450000, China

^4^School of Chemical Engineering, University of Chinese Academy of Sciences, Beijing 100049, China





**Figure S1.** (a) Temperature, (b) pressure, (c) experimental CO_2_ solubility distribution.





**Figure S2.** (a) Temperature, (b) pressure, (c) experimental N_2_ solubility distribution.





**Figure S3.** Solubility of CO_2_ in ILs measured experimentally and predicted by COSMO-RS under the same conditions.

_

_

**Figure S4.** Solubility of N_2_ in ILs measured experimentally and predicted by COSMO-RS under the same conditions.





**Figure S5.** The solubility of N_2_ in [HMIM][eFAP] measured experimentally and predicted by COSMO-RS under different pressures and the AAD between the experimental and predicted solubility at T = 303.4 K.





**Figure S6.** The relationship between temperature, pressure COSMO-RS predicted CO_2_ solubility and experimentally measured CO_2_ solubility.





**Figure S7.** The relationship between temperature, pressure, COSMO-RS predicted N_2_ solubility and experimentally measured N_2_ solubility.
